# Biofeedback Core Exercise Using Hybrid Assistive Limb for Physical Frailty Patients With or Without Parkinson's Disease

**DOI:** 10.3389/fneur.2020.00215

**Published:** 2020-04-09

**Authors:** Naoya Kotani, Takashi Morishita, Aya Yatsugi, Shinsuke Fujioka, Satoshi Kamada, Etsuji Shiota, Yoshio Tsuboi, Tooru Inoue

**Affiliations:** ^1^Department of Neurosurgery, Fukuoka University Faculty of Medicine, Fukuoka, Japan; ^2^Department of Rehabilitation Medicine, Fukuoka University Hospital, Fukuoka, Japan; ^3^Department of Neurology, Fukuoka University Faculty of Medicine, Fukuoka, Japan

**Keywords:** arthrogenic muscle inhibition, biofeedback, central pattern generator, frailty, hybrid assistive limb, Parkinson's disease

## Abstract

**Introduction:** Elderly people often exhibit “frailty,” and motor dysfunction occurs. Several studies have reported about the relationship between motor dysfunction and frailty in Parkinson's disease (PD). This study aimed to test whether the core exercise using the hybrid assistive limb lumbar type for care support (HAL-CB02) may improve the motor functions in frailty patients with or without PD and to explore the optimal patient selection from the frailty cohort.

**Materials and Methods:** We recruited 16 frailty patients (PD = 8; non-PD = 8). The participants performed core exercise and squats using HAL-CB02 for five sessions a week. Outcome measures were 10-m walking test, step length, timed up-and-go test, 30-s chair stand test, and visual analog scale. Evaluation was conducted at baseline, post-exercise, and 1- and 3-month follow-ups.

**Results:** Both PD and non-PD patients showed significant improvement in all evaluation items post-exercise. Moreover, no significant difference was found in the improvement value between the two groups.

**Conclusions:** Our results suggest that biofeedback exercise with HAL-CB02 is a safe and promising treatment for frailty patients. Motor dysfunction in PD patients may be partly due to physical frailty, and biofeedback exercise with HAL-CB02 is proposed as a treatment option.

## Introduction

The proportion of elderly people aged 65 years or older has exceeded 15% in developed countries, and it is expected to exceed 30% in 2050 (1). Physiological performance gradually decreases with aging, and frailty would be a severe burden in this population. Frailty affects activities of daily living (ADLs) and quality of life, resulting in frequent falls and walking problems. In addition, frailty is associated with mental and psychological problems, such as cognitive dysfunction and depression (2, 3). In recent years, several studies have reported on the relationship between motor dysfunction and frailty in Parkinson's disease (PD) (4–7). PD patients are likely to have frailty, and such patients are more prone to gait and balance problems than normal PD patients (5, 6).

Gait disturbance is a common problem among PD patients, and physical frailty is potentially attributable to the gait problem in PD. Atrophy and disability of erector spinae muscles have been reported to cause gait disturbance (7, 8). Trunk muscle activity plays an important role in stabilizing gait. In particular, the strength of the erector spinae muscles is highly correlated with physical activity levels (9). When the trunk leans forward during walking, a decrease in step length and an increase in cadence are observed (10). In addition, the strength of the erector spinae muscles is reduced in the leaning posture, resulting in reduced walking speed and a wide base of walking (11).

Chronic muscle disuse in physical frailty is associated with neuromuscular disorders including PD, especially in the elderly population. In contrast, resistance training is effective, but these active adaptations could not be achieved with neuromuscular electrical stimulation or traditional rehabilitation efforts alone (6, 12); thus, establishment of new treatment methods has been expected.

In the field of neurorehabilitation, the hybrid assistive limb (HAL; Cyberdyne Inc., Tsukuba, Japan) has been receiving growing attention. HAL is a robotic exoskeleton designed to facilitate movements and was developed based on the “interactive biofeedback” (iBF) hypothesis (13). Specifically, the movement of the robot is triggered by bioelectric signals (BESs) detected by surface electrodes, supporting spontaneous movement of impaired muscles generating sensory feedback. Several studies have demonstrated the efficacy and feasibility of HAL and single-joint HAL for select neurological disorders (13, 14). In this study, we used a model called HAL lumbar type for care support (HAL-CB02).

**Figure 1:**
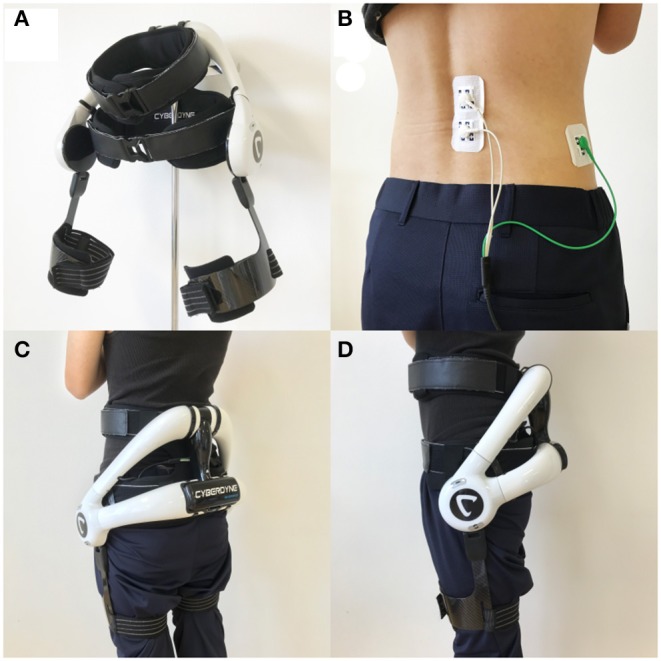
Overview of HAL-CB02. **(A)** Overall picture of HAL-CB02. **(B)** The location of electrode detecting BES from the erector spinae muscles (dual white code), and the reference electrode is at the side (single green code). **(C,D)** Back and side views of the HAL-CB02 when fully attached. BES, bioelectric signals.

HAL-CB02 is designed to mitigate risks of back pain by reducing the stress that will be applied on the back. HAL-CB02 consists of an exoskeleton frame and a power unit. The exoskeleton frame is composed of molds and belts for attachment to the lower back and the thigh and incorporates a three-axis accelerometer for measuring the absolute angle of the torso of the wearer. The power unit is composed of angle sensors and actuators of both hip joints. BES is detected from the surface electrode affixed to the erector spinae muscles; when the hip joints shift from flexion to extension, the actuator generates torque in accordance with the activity of the erector spinae muscles. The generated torque is transmitted to the wearer through the exoskeleton frame and supports standing, lifting operation, etc. By adjusting the assistance level, HAL-CB02 provides support according to the difficulty level of the movement, and the burden on the lumbar is reduced. HAL-CB02 is lightweight, as it weighs 3.1 kg including its battery, and it is easy to assemble and operate. An overview of HAL-CB02 is shown in Figure 1.

In a study using HAL for lumbar support (prototype of HAL-CB02), stress on the lumbar intervertebral disc during weight lifting was reduced (15). In addition, when HAL-CB02 was worn for lifting movements and snow shoveling, lumbar fatigue was significantly reduced and working efficiency was significantly improved (16, 17). In this context, we hypothesized that exercise with the assistance of HAL-CB02 would enable repetitive movements of core muscles under a reduced load and thus improve motor dysfunction associated with walking ability in frailty patients. We also considered that frailty patients would have muscle disuse and loss of muscle coordination in common regardless of coexistence of neurodegenerative diseases, and therefore, a robot-assisted core exercise regimen may be applied for patients with advanced PD that is often complicated with frailty. To address this hypothesis, we considered that comparing the response to the robot-assisted rehabilitation between frailty patients with and without PD is important to shed light on the relationship between frailty and PD. In this study, we aimed to test whether the core exercise and squats using the HAL-CB02 may improve the motor functions of the lower limb in frailty patients and to explore the optimal patient selection from the frailty cohort.

## Materials and Methods

### Study Design

We included elderly frailty patients with or without PD who experienced walking disability from the period between June 2017 and September 2019. In this study, frailty was diagnosed based on the definition of Fried et al. (Table 1). Frailty is diagnosed when three or more conditions in the criteria are met, while pre-frailty meets one or two conditions. In this study, we made diagnosis of the walking disability based on the self-report and the 10-m walking test (10 MWT) results showing approximately 10 s or longer. For the non-PD cohort, we included patients with frailty associated with lumbar spine problems such as lumbar canal stenosis and compression fracture. For the PD cohort, we included advanced PD patients at Hoehn and Yahr (H&Y) Stages III and IV in the on-medication state. All PD patients had been diagnosed and followed by movement disorders specialists (SF and YT). We excluded patients with severe dementia, acute bone fracture, spine problems requiring surgical treatment, severe cardiopulmonary diseases, and a physique to which the robot does not fit. We also excluded PD patients at H&Y Stage V and with severe dyskinesia.

**Table 1:** Frailty-defining criteria.

**Criteria**	**Measurement**
Weight loss	Lost >5 kg unintentionally in prior 12 months
Exhaustion	Felt exhausted for no reason in last week (self-report)
Low physical activity	Activity scale Male: <383 kcal/week Female: <270 kcal/week
Slowness	Time >10 s to walk 10 m at usual pace
Weakness	Grip strength Male: <26 kg Female: <18 kg

*Frailty: three or more criteria present*.

*Pre-frailty: one or two criteria present*.

*Robust: no criteria present*.

This prospective study was approved by our institutional review board, and informed consent was obtained from study subjects. Since this is the first report to test the feasibility of rehabilitation program using the HAL-CB02 for frailty cohorts, we included only limited numbers of patients.

**Figure 2:**
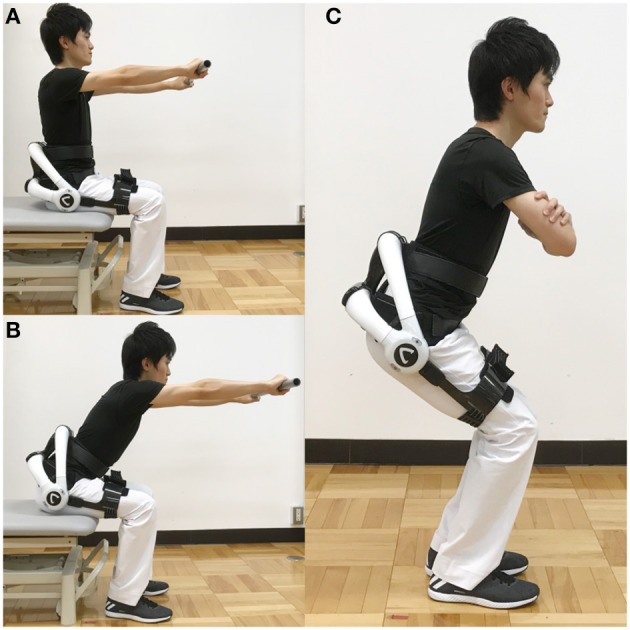
HAL-assisted exercise. **(A,B)** In the core exercise, patients were instructed to repeat bend over **(B)** and upright **(A)** positions, with the upper body in a sitting position with a pole held by extended arms. **(C)** Squat exercise with the HAL. He is a staff of our hospital, and written informed consent was obtained for publication of this study and accompanying images. HAL, hybrid assistive limb.

All patients performed five sessions of exercise using HAL-CB02. Exercise for PD patients was performed with “on” medication. The exercise time was 20–30 min per session, and participants took a rest as needed. As core exercises, pelvic tilt and forward reach were performed 30 times each. Exercises involved awareness of the anteversion of the pelvis at the sitting position and stimulation of the erector spinae muscles. In the squat method, the feet were spread apart according to the width of the shoulder, and the angle from the heel to the feet was approximately 30°. Then, the participants slowly bend their knees so that the buttocks protrude backwards, being careful that the knees are within the toe level. The knee flexion angle is targeted for a half squat (90°), and if there is knee pain, quarter squats (45°) are allowed. Then, the participants slowly extend their knees and return to the standing position. The assist level of HAL-CB02 was adjusted according to the physical state of the participants. We allowed participants with low physical function to use handrails. The number of squats was not specified, and participants were allowed to perform squats until exhaustion. The states of exercises are shown in Figure 2.

To evaluate the efficacy of the exercise, we measured physical functions using the 10 MWT, step length, timed up-and-go (TUG) test, and 30 s chair stand test (CST-30), at four time points: baseline, following five exercise sessions, 1-, and 3-month follow-up. During gait evaluation, physical therapists support the patients to prevent falls as needed. In CST-30, the participants performed sit-to-stand movements from a chair completed with arms crossed over the chest and as many times as possible within 30 s. We measured pain levels using the visual analog scale (VAS) and assess whether pain does not occur with exercise. Participants performed core exercises and squats using HAL-CB02 for five sessions within 1 week. All PD patients were also evaluated “on” medication. Adverse events associated with robot rehabilitation were also recorded such as skin problems, exacerbation of pain, and muscle damage.

### Statistical Analysis

Scores at baseline, immediately after HAL-assisted exercise, and at 1- and 3-month follow-up were compared using Friedman's test and Wilcoxon signed-rank test for intragroup comparisons. For intergroup comparisons, the Mann–Whitney *U* test was used to compare the improvement rate from baseline. Values are presented as mean ± standard deviation. SPSS 24.0 (IBM Corp., Armonk, NY, USA) was used for statistical analysis. A *p* < 0.05 was considered significant. We also performed Friedman's test to test the null hypothesis of no change in the number of squats during the training period.

## Results

**Figure 3:**
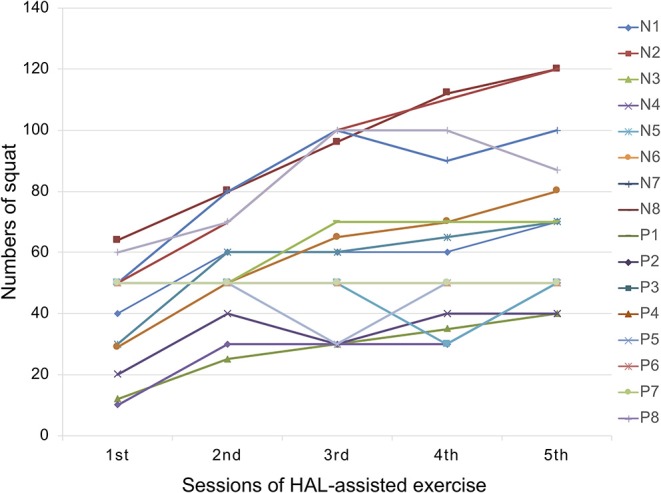
Transition graph showing number of squats at each session. HAL, hybrid assistive limb; N, non-Parkinson's disease; P, Parkinson's disease.

We recruited 16 frailty patients including eight non-PD and eight PD patients. Baseline demographics are summarized in Tables 2, 3. No significant differences in any demographic features were found between the two groups. All non-PD patients had a history of some chronic spine problems inclusive of lumbar canal stenosis (*n* = 5), vertebral compression fracture (*n* = 2), and spina bifida (*n* = 1). Peak dose dyskinesia potentially affecting robot-assisted exercise program was not observed in all PD participants. In the PD group, 1- and 3-month follow-up data could only be evaluated in five and four patients, respectively, due to accessibility to the follow-up clinic. All participants completed the HAL-assisted exercise successfully, without any adverse events, and the squat frequency increased significantly with each session (*p* < 0.001) (Figure 3).

**Table 2:** Baseline demographics of two cohorts.

	**Non-PD**	**PD**	***p*-values**
*N*	8	8	
Age (years)	73.8 ± 13.2	68.6 ± 8.3	0.161
Sex	Male 3 (37.5%)	Male 4 (50.0%)	0.614
	Female 5 (62.5%)	Female 4 (50.0%)	
Disease duration (PD, years)	N/A	10.9 ± 7.1	
H&Y stage (PD)	N/A	III 3 (37.5%) IV 5 (62.5%)	
Weight (kg)	58.0 ± 9.1	56.3 ± 13.8	0.959
Height (cm)	158.3 ± 8.9	159.9 ± 13.0	1.000
BMI	23.1 ± 2.4	21.8 ± 4.0	0.279
10 MWT (s)	35.5 ± 31.1	20.3 ± 13.3	0.328
Step length (m)	0.33 ± 0.13	0.38 ± 0.16	0.645
TUG (s)	37.9 ± 30.9	19.5 ± 8.5	0.279
CST-30 (times)	4.0 ± 3.7	3.3 ± 2.2	0.645

*10 MWT, 10 m walking test; BMI, body mass index; CST-30, 30 s chair stand test; H&Y stage, Hoehn and Yahr Stage; PD, Parkinson's disease; TUG, timed up and go test*.

*Measured values are presented as means ± standard deviation*.

**Table 3:** Patient characteristics.

**Non-PD group**					**PD group**				
**Case**	**Age**	**Sex**	**Frailty**	**Comorbid spine problems**	**Case**	**Age**	**Sex**	**Frailty**	**Comorbid spine problems**	**H&Y stage (on/off)**
1	84	F	Frailty	Mild LCS	1	75	F	Frailty	Lumbar spondylosis (L4,5)	III/IV
2	79	M	Pre-frailty	LCS s/p laminectomy	2	63	F	Pre-frailty	None	IV/IV
3	87	F	Frailty	LCS s/p PLIF	3	65	F	Pre-frailty	None	III/III
4	46	F	Pre-frailty	Spina bifida	4	61	M	Pre-frailty	None	IV/IV
5	73	F	Frailty	Mild LCS	5	60	M	Pre-frailty	None	IV/IV
6	67	M	Pre-frailty	Vertebral compression fx (L2)	6	66	M	Pre-frailty	Mild LCS	IV/IV
7	83	F	Pre-frailty	Vertebral compression fx (L5)	7	76	F	Frailty	None	IV/IV
8	71	M	Pre-frailty	LCS s/p PLIF	8	83	M	Frailty	Mild LCS	III/III
	73.8 ± 13.2	3 males 5 females				68.6 ± 8.3	4 males 4 females			

*fx, fracture; H&Y stage, Hoehn and Yahr stage; LCS, lumbar canal stenosis; PD, Parkinson's disease; PLIF, posterior lumbar interbody fusion; s/p, status post*.

**Figure 4:**
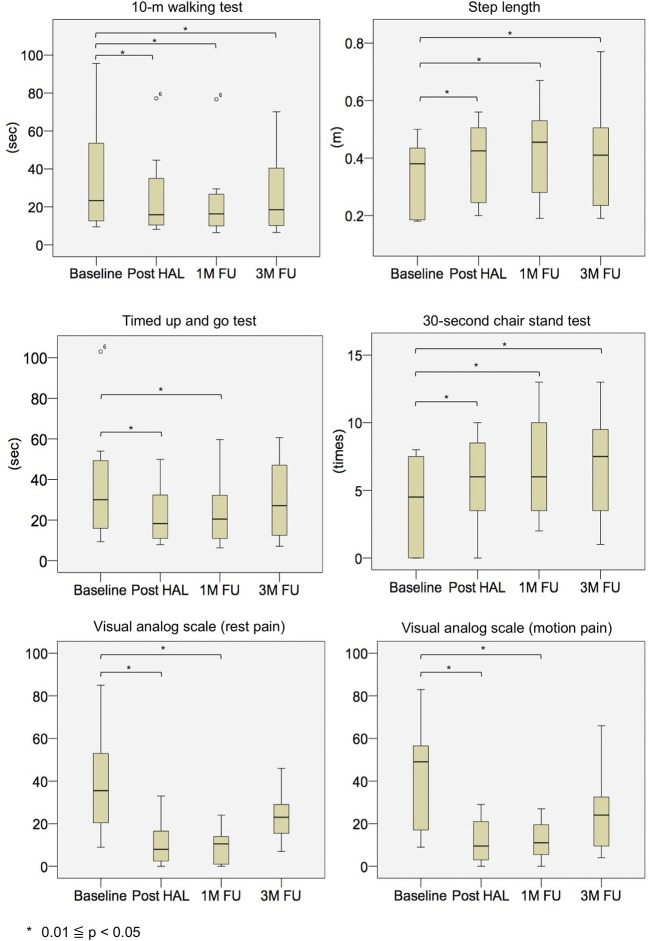
Box plots depicting outcome measures in the non-PD group at baseline, post-HAL, and at 1- and 3-month follow-ups. FU, follow-up; HAL, hybrid assistive limb; PD, Parkinson's disease.

Physical evaluations showed significant improvements. The measured median and interquartile range values (*p*-values compared with baseline) from the evaluation items of the non-PD group at baseline, post-HAL, and 1- and 3- month follow-up were as follows: 10 MWT values were 23.3 [13.5, 46.3] s at baseline, 15.9 [10.8, 30.2] s (*p* = 0.012) post-HAL, 16.3 [10.2, 25.3] s (*p* = 0.012) at 1-month follow-up, and 18.5 [10.3, 34.6] s (*p* = 0.012) at 3-month follow-up. Step length values were 0.38 [0.19, 0.43] m at baseline, 0.43 [0.26, 0.49] m (*p* = 0.012) post-HAL, 0.46 [0.32, 0.53] m (*p* = 0.012) at 1-month follow-up, and 0.41 [0.24, 0.49] m (*p* = 0.012) at 3-month follow-up. TUG values were 30.1 [17.5, 47.0] s at baseline, 18.3 [11.3, 28.8] s (*p* = 0.012) post-HAL, 20.5 [11.0, 31.6] s (*p* = 0.012) at 1-month follow-up, and 27.1 [13.1, 42.6] s (*p* = 0.093) at 3-month follow-up. CST-30 values were 4.5 [0.0, 7.3] times at baseline, 6.0 [3.8, 8.3] times (*p* = 0.017) post-HAL, 6.0 [3.8, 9.5] times (*p* = 0.011) at 1-month follow-up, and 7.5 [3.8, 8.8] times (*p* = 0.024) at 3-month follow-up (Figure 4 and Table 4). In addition, three participants with frailty at baseline improved to pre-frailty at 1-month follow-up, and two of them were able to keep up even at 3-month follow-up. Also, two of the five participants with pre-frailty at baseline were “robust” at 1-month follow-up, and they maintained this state even at 3-month follow-up.

**Table 4:** Details of clinical outcomes.

	**Non-PD group**				**PD group**			
	**Baseline**	**Post HAL**	**1 M follow-up**	**3 M follow-up**	**Baseline**	**Post HAL**	**1 M follow-up**	**3 M follow-up**
10 MWT (s)	23.3 [13.5, 46.3]	15.9 [10.8, 30.2] (0.012)	16.3 [10.2, 25.3] (0.012)	18.5 [10.3, 34.6] (0.012)	15.3 [10.6, 26.7]	9.6 [8.5, 13.3] (<0.001)	12.0 [9.4, 13.8] (0.001)	10.4 [10.1, 10.9] (0.006)
Step length (m)	0.38 [0.19, 0.43]	0.43 [0.26, 0.49] (0.012)	0.46 [0.32, 0.53] (0.012)	0.41 [0.24, 0.49] (0.012)	0.37 [0.28, 0.47]	0.51 [0.42, 0.60] (<0.001)	0.42 [0.40, 0.48] (0.001)	0.52 [0.47, 0.57] (0.003)
TUG (s)	30.1 [17.5, 47.0]	18.3 [11.3, 28.8] (0.012)	20.5 [11.0, 31.6] (0.012)	27.1 [13.1, 42.6] (0.093)	17.7 [12.9, 22.7]	14.0 [10.1, 20.2] (<0.001)	14.6 [11.5, 17.8] (0.002)	11.7 [11.5, 18.3] (0.136)
CST-30 (times)	4.5 [0.0, 7.3]	6.0 [3.8, 8.3] (0.017)	6.0 [3.8, 9.5] (0.011)	7.5 [3.8, 8.8] (0.024)	4.0 [2.3, 4.3]	6.5 [5.8, 8.3] (0.001)	7.0 [7.0, 9.0] (0.001)	9.0 [6.8, 11.8] (0.006)
VAS at rest	35.5 [23.3, 48.5]	8.0 [3.8, 16.3] (0.036)	10.5 [1.5, 14.0] (0.012)	23.0 [17.8, 28.5] (0.233)				
VAS in motion	49.0 [19.5, 55.3]	9.5 [4.5, 18.5] (0.017)	11.0 [5.8, 17.8] (0.028)	24.0 [10.8, 31.8] (0.176)				

*10 MWT, 10 m walking test; CST-30, 30 s chair stand test; HAL, hybrid assistive limb; PD, Parkinson's disease; TUG, timed up-and-go test; VAS, visual analog scale*.

*Measured values are presented as median [interquartile range] and (p-values compared to baseline)*.

**Figure 5:**
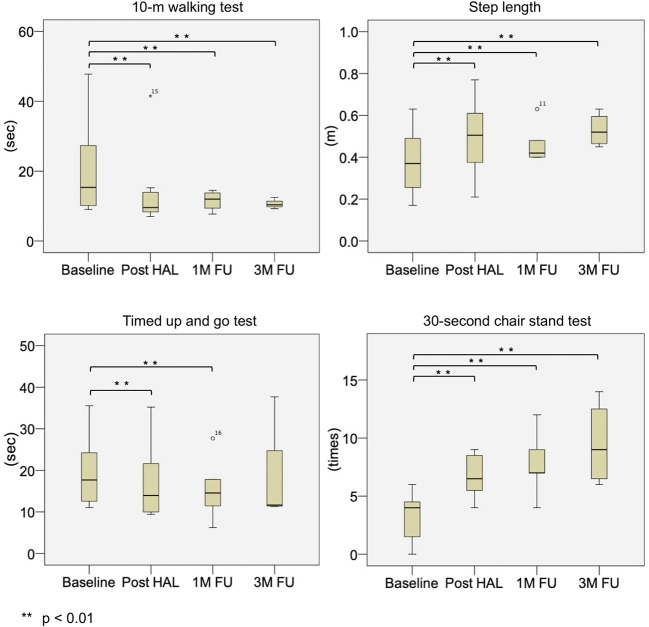
Box plots depicting outcome measures in the PD group at baseline, post-HAL, and at 1- and 3-month follow-ups. FU, follow-up; HAL, hybrid assistive limb; PD, Parkinson's disease.

The measured median and interquartile range values (*p*-values compared with baseline) from the evaluation items of the PD group at baseline, post-HAL, and 1- and 3- month follow-ups were as follows: 10 MWT values were 15.3 [10.6, 26.7] s at baseline, 9.6 [8.5, 13.3] s (*p* < 0.001) post-HAL, 12.0 [9.4, 13.8] s (*p* = 0.001) at 1-month follow-up, and 10.4 [10.1, 10.9] s (*p* = 0.006) at 3-month follow-up. Step length values were 0.37 [0.28, 0.47] m at baseline, 0.51 [0.42, 0.60] m (*p* < 0.001) post-HAL, 0.42 [0.40, 0.48] m (*p* = 0.001) at 1-month follow-up, and 0.52 [0.47, 0.57] m (*p* = 0.003) at 3-month follow-up. TUG values were 17.7 [12.9, 22.7] s at baseline, 14.0 [10.1, 20.2] s (*p* < 0.001) post-HAL, 14.6 [11.5, 17.8] s (*p* = 0.002) at 1-month follow-up, and 11.7 [11.5, 18.3] s (*p* = 0.136) at 3-month follow-up. CST-30 values were 4.0 [2.3, 4.3] times at baseline, 6.5 [5.8, 8.3] times (*p* = 0.001) post-HAL, 7.0 [7.0, 9.0] times (*p* = 0.001) at 1-month follow-up, and 9.0 [6.8, 11.8] times (*p* = 0.006) at 3-month follow-up (Figure 5 and Table 4). In addition, two participants with frailty at baseline improved to pre-frailty at 1-month follow-up, and one of them was able to keep up even at 3-month follow-up. Also, two of the six participants with pre-frailty at baseline were “robust” at 1-month follow-up, and one of them maintained this state even at 3-month follow-up.

**Figure 6:**
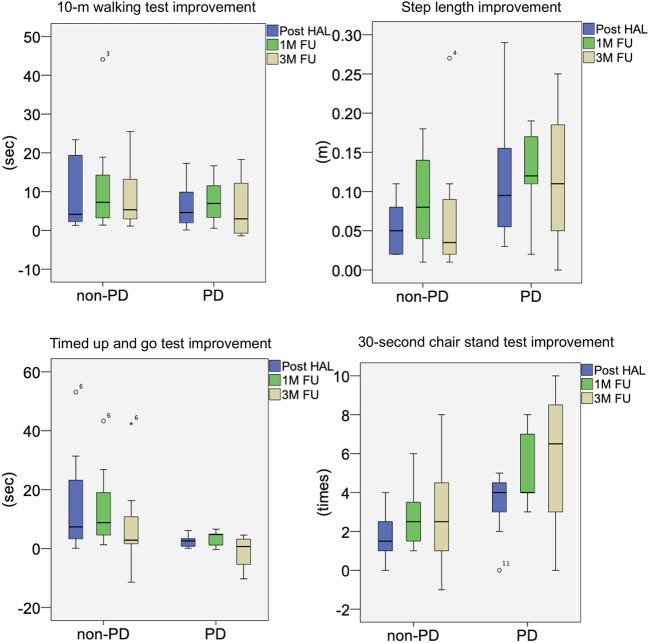
Box plots of intergroup comparison of improvement rates from baseline for non-PD and PD groups. FU, follow-up; HAL, hybrid assistive limb; PD, Parkinson's disease.

Moreover, the improvement values from the baseline of each evaluation item in the non-PD group and PD group were compared. In all evaluation items, significant differences between the two groups at all time points were not observed (Figure 6).

Pain levels were reduced with HAL-assisted exercise. All patients in the non-PD group had low back pain, but post-HAL, the pain was significantly reduced and the effect persisted even after 1-month follow-up; however, at 3-month follow-up, a statistically significant difference was not observed, even if the measured value was higher than the baseline. In the PD group, no patients complained of low back pain, and pain related to HAL-assisted exercise was not reported. In the non-PD group, measured median and interquartile range values (*p*-values compared with baseline) of VAS score at rest and in motion at baseline, post-HAL, at 1- and 3-month follow-up were as follows: VAS scores at rest were 35.5 [23.3, 48.5] at baseline, 8.0 [3.8, 16.3] (*p* = 0.036) post-HAL, 10.5 [1.5, 14.0] (*p* = 0.012) at 1-month follow-up, and 23.0 [17.8, 28.5] (*p* = 0.233) at 3-month follow-up. VAS scores in motion were 49.0 [19.5, 55.3] at baseline, 9.5 [4.5, 18.5] (*p* = 0.017) post-HAL, 11.0 [5.8, 17.8] (*p* = 0.028) at 1-month follow-up, and 24.0 [10.8, 31.8] (*p* = 0.176) at 3-month follow-up (Figure 4 and Table 4).

## Discussion

To the best of our knowledge, this study is the first to use HAL-CB02 in frailty and PD patients. HAL-CB02 may improve motor function. This result has the potential to improve frailty from a long-term perspective and clarified the feasibility of HAL-assisted exercise. One advantage of the robot rehabilitation is that the robot enables repeated performance of the same movements that are usually difficult to assist manually. We speculate that robot rehabilitation improves motor coordination by controlling axial muscles.

There are several reports of robot-assisted gait training (RAGT) for PD. Cappecci et al. reported that RAGT significantly improved endurance, gait capacity, motor symptoms, quality of life, and freezing gait (18). In addition, Alwardat et al.'s meta-analysis reported that RAGT showed better outcomes than conventional interventions in some motor aspects of PD (19). Robot-assisted rehabilitation enables standardized treatment regardless of the therapists' experience and repetitive exercise without patient's fatigue as shown in our results. Most of the reported RAGTs are based on gait assist robots, but we anticipate that HAL-CB02, as a treatment with core exercise and squats, can be performed more easily and safely. Concerning the similar improvements in two cohorts in our study, there are several explanations.

**Figure 7:**
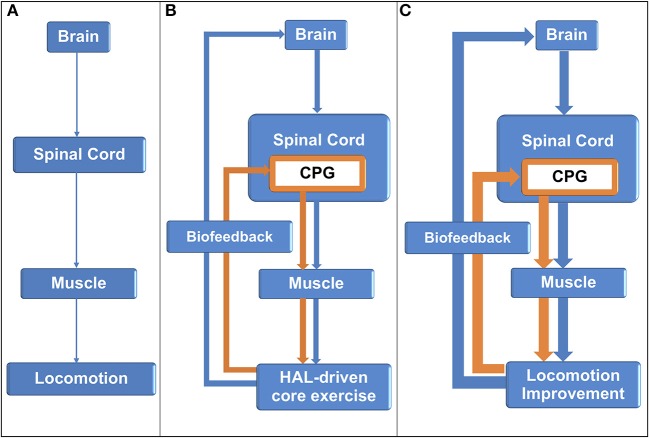
Central nervous system activation by sensory feedback from hybrid assistive limb-assisted training. **(A)** Central nervous system (CNS) lesion resulted in gait disability. **(B)** The hybrid assistive limb (HAL) assisted core function, and sensory input was sent back to the CNS levels to activate the brain and the central pattern generator in the spinal cord. **(C)** In turn, the damaged CNS generated improved descending signals to the muscle for better locomotion.

In this study, both PD and non-PD patients showed significantly improved motor function. In addition, since no significant difference was found between these two groups in terms of the improvement rate, it is expected that patients with physical frailty may have the same motor dysfunction regardless of the presence or absence of PD. From the standing point, we consider that the disturbance of the central pattern generator (CPG) in the spinal cord exists in common among frailty patients. Repetitive sensory feedback from HAL training may activate the central nervous system (CNS) and possibly induce neuroplasticity in the spinal cord level to facilitate functional recovery in the disused neuronal networks (20) (Figure 7).

Although there may be common factors for improvement among non-PD and PD cohorts, another factor may contribute to the improvement differently. We speculate that arthrogenic muscle inhibition (AMI) may be also related as a cause of failure of conventional rehabilitation of frailty patients, especially in a non-PD cohort with back pain. AMI is defined as the suppression of motor neurons due to trauma and the associated pain, resulting in decreasing muscle function. It is thought that abnormality of proprioceptive receptors due to swelling, inflammation, pain, and joint laxity causes AMI (21, 22). AMI is a reflexive response that acts as a protective mechanism to prevent further damage to the joint (23). AMI is the result of many different joint receptor activities. It acts on inhibitory interneurons that form synapses in the motor neuron pool of articular muscle tissue (24). Rice et al. proposed three spinal reflex pathways related to AMI: the group I non-reciprocal (Ib) inhibitory pathway, flexion reflex, and gamma (γ)-loop. When abnormality occurs in the peripheral joints and changes the afferent discharge from proprioceptive receptors, these spinal reflex pathways are impaired (21). Furthermore, joint afferents are susceptible to changes in discharge (25, 26), and the spinal descending pathway may strongly influence interneurons and motor neurons at the spinal level (27–29). Several studies have described the relationship between spinal reflex and AMI (21, 30), and proprioceptive sensory feedback is related to reflex inhibition (31, 32). Although AMI was reported to be related to lower-limb functions in many cases, Russo et al. reported that AMI of paravertebral muscles was easily affected by damage to the lumbar region (33). As described above, it is speculated that physical frailty patients easily develop neuromuscular disorders and are prone to dysfunction of the erector spinae muscles, and they are likely to have AMI.

An effective treatment for AMI includes biofeedback therapy (34, 35). Most of the reports are based on electromyographic biofeedback, which measures the electrical activity of the muscle from the electrodes attached to the skin surface and feeds back the magnitude of the muscle activity visually and auditorily (36–41). Similarly, in this study, we consider that biofeedback with HAL-CB02 had improved AMI. We considered that HAL-assisted exercise stimulates proper proprioceptive receptors by repeatedly feeding back correct motion at low load and suppresses abnormal spinal reflexes. Actually, our group has shown the possibility of AMI improvement by HAL-assisted exercise in patients who underwent total knee arthroplasty (42, 43). Similarly, our non-PD patients showed significant pain reduction following HAL-assisted exercise, and this may partly contribute to the improvement in motor functions.

As limitations of this study, we did not evaluate ADL, quality of life, and objective measures such as electromyograms, so we could not identify the clinical impact and cause of improvement. Since the subjects with only exercise without HAL-CB02 were not recruited as control, we could not measure the efficacy of the robot-assisted exercise. Furthermore, the sample size was small, and several patients in the PD group were unable to complete follow-up evaluation. Future investigation on these issues with an increased number of cases is necessary to confirm our findings.

## Conclusions

Our results suggest that biofeedback therapy with HAL-CB02 may be a safe and promising treatment for patients with physical frailty even complicated with spine problems. In addition, motor dysfunction in PD patients may be partly due to physical frailty, and biofeedback therapy with HAL-CB02 is proposed as a treatment option. Immediate and sustained effects on patients who were refractory to conventional rehabilitation could provide evidence that changes in input to specific receptors by HAL-CB02 contribute to activation of disused neuronal networks and amelioration of AMI. Further long-term follow-up studies with an increased number and control cohort of conventional rehabilitation are warranted.

## Data Availability Statement

The datasets generated for this study are available on request to the corresponding author.

## Ethics Statement

The studies involving human participants were reviewed and approved by Fukuoka university institutional review board. The patients/participants provided their written informed consent to participate in this study.

## Author Contributions

NK, TM, and TI: concept and design. TM, SF, and YT: acquisition of subjects. NK and AY: acquisition of data. NK, TM, SK, and ES: interpretation of data. NK, TM, SF, SK, ES, YT, and TI: preparation of manuscript.

### Conflict of Interest

The authors declare that the research was conducted in the absence of any commercial or financial relationships that could be construed as a potential conflict of interest.

## References

[1] He W Goodkind D Kowal P An aging world : 2015 international population reports. Aging. (2016) 165. Available online at: https://www.census.gov/content/dam/Census/library/publications/2016/demo/p95-16-1.pdf

[2] Fried LP Tangen CM Walston J Newman AB Hirsch C Gottdiener J. Frailty in older adults: evidence for a phenotype. J Gerontol Med Sci Am. (2001) 56:146–57. 10.1093/gerona/56.3.M146 11253156

[3] Clegg A Young J Iliffe S Rikkert MO Rockwood K. Frailty in elderly people. Lancet. 381:752–62. 10.1016/S0140-6736(12)62167-9 23395245 PMC4098658

[4] Smith N Brennan L Gaunt DM Ben-Shlomo Y Henderson E. Frailty in parkinson's disease: a systematic review. J Parkinsons Dis. (2019) 9:517–24. 10.3233/JPD-191604 31381530

[5] Peball M Mahlknecht P Werkmann M Marini K Murr F Herzmann H. Prevalence and associated factors of sarcopenia and frailty in Parkinson's disease: a cross-sectional study. Gerontology. (2019) 65:216–28. 10.1159/000492572 30199864

[6] Müller MLTM Marusic U van Emde Boas M Weiss D Bohnen NI. Treatment options for postural instability and gait difficulties in Parkinson's disease. Expert Rev Neurother. (2019) 19:1229–51. 10.1080/14737175.2019.1656067 31418599

[7] Chiang C-K Chen H-L Lin C-H Chen M-H Chiang P-L Chen Y-S. Altered body composition of psoas and thigh muscles in relation to frailty and severity of parkinson's disease. Int J Environ Res Public Health. (2019) 16:3667. 10.3390/ijerph16193667 31569569 PMC6801975

[8] Crawford R Gizzi L Dieterich A Mhuiris ÁN Falla D. Age-related changes in trunk muscle activity and spinal and lower limb kinematics during gait. PLoS ONE. (2018) 13:1–15. 10.1371/journal.pone.0206514 30408111 PMC6224053

[9] Sinaki M Offord KP. Physical activity in postmenopausal women: effect on back muscle strength and bone mineral density of the spine. Arch Phys Med Rehabil. (1988) 69:277–80. 3258510

[10] Saha D Gard S Fatone S. The effect of trunk flexion on able-bodied gait. Gait Posture. (2008) 27:653–60. 10.1016/j.gaitpost.2007.08.009 17920272

[11] Balzini L Vannucchi L Benvenuti F Benucci M Monni M Cappozzo A. Clinical characteristics of flexed posture in elderly women. J Am Geriatr Soc. (2003) 51:1419–26. 10.1046/j.1532-5415.2003.51460.x 14511162

[12] Suetta C. Plasticity and function of human skeletal muscle in relation to disuse and rehabilitation: influence of ageing and surgery. Dan Med J. (2017) 64:B5377. Available online at: https://ugeskriftet.dk/dmj/plasticity-and-function-human-skeletal-muscle-relation-disuse-and-rehabilitation-influence-ageing 28869034

[13] Morishita T Inoue T. Interactive bio-feedback therapy using hybrid assistive limbs for motor recovery after stroke: current practice and future perspectives. Neurol Med Chir. (2016) 56:605–12. 10.2176/nmc.st.2016-0094 27616320 PMC5066081

[14] Fukuda H Morishita T Ogata T Saita K Hyakutake K Watanabe J. Tailor-made rehabilitation approach using multiple types of hybrid assistive limb robots for acute stroke patients: a pilot study. Assist Technol. (2016) 28:53–6. 10.1080/10400435.2015.1080768 26478988

[15] Hara H Sankai Y Evaluation of HAL for lumbar support by 3D skeletal model. Trans Japanese Soc Med Biol Eng. (2012) 50:111–16. 10.11239/jsmbe.50.111

[16] Miura K Kadone H Koda M Abe T Kumagai H Nagashima K. The hybrid assistive limb (HAL) for care support successfully reduced lumbar load in repetitive lifting movements. J Clin Neurosci. (2018) 53:276–9. 10.1016/j.jocn.2018.04.057 29731278

[17] Miura K Kadone H Koda M Abe T Endo H Murakami H. The hybrid assisted limb (HAL) for care support, a motion assisting robot providing exoskeletal lumbar support, can potentially reduce lumbar load in repetitive snow-shoveling movements. J Clin Neurosci. (2017) 49:83–6. 10.1016/j.jocn.2017.11.020 29254733

[18] Capecci M Pournajaf S Galafate D Sale P Le Pera D Goffredo M. Clinical effects of robot-assisted gait training and treadmill training for Parkinson's disease. A randomized controlled trial. Ann Phys Rehabil Med. (2019) 62:303–12. 10.1016/j.rehab.2019.06.016 31377382

[19] Alwardat M Etoom M Al Dajah S Schirinzi T Di Lazzaro G Salimei PS. Effectiveness of robot-assisted gait training on motor impairments in people with Parkinson's disease: a systematic review and meta-analysis. Int J Rehabil Res. (2018) 41:287–96. 10.1097/MRR.0000000000000312 30119060

[20] Yatsugi A Morishita T Fukuda H Kotani N Yagi K Abe H. Feasibility of neurorehabilitation using a hybrid assistive limb for patients who underwent spine surgery. Appl Bionics Biomech. (2018) 2018:1–11. 10.1155/2018/7435746 30116296 PMC6079604

[21] Rice DA McNair PJ. Quadriceps arthrogenic muscle inhibition: neural mechanisms and treatment perspectives. Semin Arthritis Rheum. (2010) 40:250–66. 10.1016/j.semarthrit.2009.10.001 19954822

[22] Palmieri RM Ingersoll CD Hoffman MA Cordova ML Porter DA Edwards JE. Arthrogenic muscle response to a simulated ankle joint effusion. Br J Sports Med. (2004) 38:26–30. 10.1136/bjsm.2002.001677 14751941 PMC1724745

[23] Young A. Current issues in arthrogenous inhibition. Ann Rheum Dis. (1993) 52:829–34. 10.1136/ard.52.11.829 8250616 PMC1005198

[24] Hopkins JT Ingersoll CD Arthrogenic muscle inhibition: a limiting factor in joint rehabilitation. J Sport Rehabil. (2000) 9:135–59. 10.1123/jsr.9.2.135

[25] Baumeister J Reinecke K Weiss M. Changed cortical activity after anterior cruciate ligament reconstruction in a joint position paradigm: an EEG study. Scand J Med Sci Sport. (2008) 18:473–84. 10.1111/j.1600-0838.2007.00702.x 18067525

[26] Pitman MI Nainzadeh N Menche D Gasalberti R Eun Kyoo S. The intraoperative evaluation of the neurosensory function of the anterior cruciate ligament in humans using somatosensory evoked potentials. Arthrosc J Arthrosc Relat Surg. (1992) 8:442–7. 10.1016/0749-8063(92)90005-V 1466702

[27] Schomburg ED. Spinal sensorimotor systems and their supraspinal control. Neurosci Res. (1990) 7:265–340. 10.1016/0168-0102(90)90008-3 2156196

[28] Jankowska E. Interneuronal relay in spinal pathways from proprioceptors. Prog Neurobiol. (1992) 38:335–78. 10.1016/0301-0082(92)90024-9 1315446

[29] Millan MJ. Descending control of pain. Prog Neurobiol. (2002) 66:355–474. 10.1016/S0301-0082(02)00009-6 12034378

[30] Hopkins JT Ingersoll CD Edwards J Klootwyk TE Cryotherapy and transcutaneous electric neuromuscular stimulation decrease arthrogenic muscle inhibition of the vastus medialis after knee joint effusion. J Athl Train. (2001) 2537:25–31. PMC164304 12937440

[31] Brumagne S Lysens R Swinnen S Verschueren S. Effect of paraspinal muscle vibration on position sense of the lumbosacral spine. Spine. (1999) 24:1328–31. 10.1097/00007632-199907010-00010 10404575

[32] Le Pera D Graven-Nielsen T Valeriani M Oliviero A Di Lazzaro V Tonali PA. Inhibition of motor system excitability at cortical and spinal level by tonic muscle pain. Clin Neurophysiol. (2001) 112:1633–41. 10.1016/S1388-2457(01)00631-9 11514246

[33] Russo M Deckers K Eldabe S Kiesel K Gilligan C Vieceli J. Muscle control and non-specific chronic low back pain. Neuromodulation. (2018) 21:1–9. 10.1111/ner.12738 29230905 PMC5814909

[34] Pietrosimone B McLeod MM Florea D Gribble PA Tevald MA. Immediate increases in quadriceps corticomotor excitability during an electromyography biofeedback intervention. J Electromyogr Kinesiol. (2015) 25:316–22. 10.1016/j.jelekin.2014.11.007 25561075

[35] Gabler CM Kitzman PH Mattacola CG. Targeting quadriceps inhibition with electromyographic biofeedback: a neuroplastic approach. Crit Rev Biomed Eng. (2013) 41:125–35. 10.1615/CritRevBiomedEng.2013008373 24580566

[36] Campenella B Mattacola CG Kimura IF Effect of visual feedback and verbal encouragement on concentric quadriceps and hamstrings peak torque of males and females. Isokinet Exerc Sci. (2000) 8:1–6. 10.3233/IES-2000-0033

[37] Croce RV. The effects of EMG biofeedback on strength acquisition. Biofeedback Self Regul. (1986) 11:299–310. 10.1007/BF01000166 3607096

[38] Lucca JA Recchiuti SJ. Effect of electromyographic biofeedback on an isometric strengthening program. Phys Ther. (1983) 63:200–3. 10.1093/ptj/63.2.200 6823470

[39] Kimura IF Gulick DT Gasiewski E Effect of visual feedback on concentric peak torque production during knee extension and flexion exercise in males and females. Isokinet Exerc Sci. (1997) 6:209–14. 10.3233/IES-1997-6403

[40] Davlin CD Holcomb WR Guadagnoli MA. The effect of hip position and electromyographic biofeedback training on the vastus medialis oblique: vastus lateralis ratio. J Athl Train. (1999) 34:342–6. 16558584 PMC1323342

[41] O'Sullivan A O'Sullivan K The effect of combined visual feedback and verbal encouragement on isokinetic concentric performance in healthy females. Isokinet Exerc Sci. (2008) 16:47–53. 10.3233/IES-2008-0295

[42] Goto K Morishita T Kamada S Saita K Fukuda H Shiota E. Feasibility of rehabilitation using the single-joint hybrid assistive limb to facilitate early recovery following total knee arthroplasty: a pilot study. Assist Technol. (2017) 29:197–201. 10.1080/10400435.2016.1219883 27689789

[43] Kotani N Morishita T Saita K Kamada S Maeyama A Abe H. Feasibility of supplemental robot-assisted knee flexion exercise following total knee arthroplasty. J Back Musculoskelet Rehabil. (2019) 1–9. [Epub ahead of print]. 10.3233/BMR-181482 31561326

